# The International Congress of Hygiene and Demography

**Published:** 1891-08-15

**Authors:** 


### The International Congress of Hygiene and Demography.

Demography has been roughly defined as the science of crowds, and certainly the members of this Congress, owing to the crowded condition of many of the sections and meetings have had an excellent opportunity during the past week of studying this branch of science in the most practical manner. St. James's Hall never looked better than it did on Monday afternoon when the great audience rose to greet the Prince of Wales. It is the fashion to belittle scientific congresses, especially if they relate to health, but we are convinced there never was a Congress which presented more features of interest, where the discussions were on the whole better sustained, or where the public and the profession, as apart from the official classes, whose absence from the opening meeting was a matter of general comment—have by united effort produced more successful results. The Congress has had, of course, two aspects: its scientific and its social. The scientific has excited the widest interest, as well it might, having regard to the importance of the subjects dealt with by many of the readers of papers and speakers. Amongst those of more general interest we have only room to mention the following:—

On Tuesday, Dr. F. Warner, of London, read a paper on the "Scientific observation and study of Children in Schools, and the Classes into which they may be grouped." The Rev. Canon Diggle, Dr. Fletcher Beach, Dr. Shuttleworth, and General Moberly took part in the discussion.

The following interesting papers were also read:—

Dr. Octavius Sturges, of London: "The early recognition and probable arrest of St. Vitus' Dance in School Children."

Mr. J. Jackson: "Handwriting in relation to Hygiene."

Dr. Jacobi, of New York: "The Laws regulating the Employment of Children in the United States of America."

Mr. G. Cunningham, M.A., L.D.S., of Cambridge: "The Care of Children's Teeth."

A Discussion introduced by Dr. W. J. Russell, F R.S., on "Town Fogs and their Effects, including Smoke-prevention," created some interest. An apparatus demonstrating the possibility of removing the smoke of fires from the air of towns, and of using it for disinfecting sewers, was exhibited by Mr. Sheridan Delépine, M.B., B Sc., and Mr. Alfred B. Gomess.

Common Lodging Houses: Mr. P. Gordon Smith, F.R.I.B.A., Architect to the Local Government Board.

Dr. Koch's new Institute: Herr J. Böttger, Berlin.

English Isolation Hospitals: Mr. R. Thorne Thorne, M.D., Inspector, Local Government Board.

The Heathcote Hospital, Leamington: Mr. Keith D. Young, F.R I.B A.

Hospital Tents and Huts: Dr. Duchausoy.

Asylum Arrangement and Construction: Mr. Richard Greene, M.D., and Mr. G. T. Hime, F.R.I B.A.

The social aspects of the Congress has been almost unique. Never before has Hygiene excited so much general hospitality. The Court of Common Council, under the direction of a committee of which Alderman Stuart Knill was the able Chairman, gave a most hospitable welcome to the members at the Guildhall on Tuesday evening, when upwards of three thousand invitations were issued. Mr. John Thomas, who conducted a band of lady harpists, delighted his audiences by several solos. The Baroness Burdett-Coutts gave a charming garden party on the afternoon of the same day; the day was fine, and the scene a very brilliant one. On Wednesday afternoon, the three garden parties given by Sir E. Saunders, Lady Wantage, and at the Royal Normal School for the Blind, were all well attended, in spite of the fact that the weather was somewhat unsettled throughout the day. The soirée at the Royal College of Physicians, though of a very interesting nature, had a somewhat sombre appearance, owing to the absence of the bright costumes of the ladies. Thursday was marked by the visit of a large concourse of visitors to Wormwood Scrubs prison. This afforded the foreign visitors an exceptional opportunity to gain an insight into our criminal administration. In the evening a large party assembled at the Crystal Palace to dine, and witness a display of fireworks. Mr. Brock had made great preparations for the occasion. Besides the lighter forms of entertainment, visitors were afforded ample opportunities of viewing for themselves many of the most interesting institutions of the nation, such as the London hospitals, the Naval Hospital, Netley, Buckingham Palace, Windsor Castle, Hampton Court, Gas, Iron, and other works, and the destructors and sewage farm at Birmingham. Excursions of all kinds are arranged for to-day (Saturday), and to foreign visitors especially the Congress offers every facility to become acquainted with English scientific, social, and industrial institutions. The Ladies' Committee have been especially active in arranging for the lady guests, and we think all the visitors will be satisfied with the efforts made on their behalf. In a word, the Congress of Hygiene for 1891 is proving the greatest success, and promises to leave behind it many pleasant memories.

